# Bacteriological Course for Veterinarians in Baden

**Published:** 1896-06

**Authors:** 


BACTERIOLOGICAL COURSE FOR VETERINARIANS IN BADEN. According to the announcement of the Baden Ministry of the Interior in November, 1895, a bacteriological course for veterinary surgeons will be held in January of this year in the Bacteriological Institute of the Technical High School in Karlsruhe.

The course lasts ten days, and can be attended free by the district veterinary surgeons of the Grand Duchy. A course of wider scope has for a long time been agitated by the Austrian Society of Veterinarians, and it is hoped that the friendly compliance of the professors in the Vienna Veterinary School will soon be realized. The request from the central committee has already been handed in.—*Ibid.*

